# CT-monitored minimal ablative margin control in single-session microwave ablation of liver tumors: an effective strategy for local tumor control

**DOI:** 10.1007/s00330-022-08723-5

**Published:** 2022-04-07

**Authors:** Ijin Joo, Kenneth W. Morrow, Steven S. Raman, Justin P. McWilliams, James W. Sayre, David S. Lu

**Affiliations:** 1 Department of Radiology, David Geffen School of Medicine, University of California, Los Angeles, CA USA; 2 Department of Radiology, Seoul National University Hospital and Seoul National University College of Medicine, Seoul, South Korea

**Keywords:** Ablation techniques, Microwaves, Computed tomography, X-ray, Liver neoplasms

## Abstract

**Objectives:**

To investigate the usefulness of minimal ablative margin (MAM) control by intra-procedural contrast-enhanced CT (CECT) in microwave ablation (MWA) of liver tumors.

**Methods:**

A total of 334 consecutive liver tumors (240 hepatocellular carcinomas [HCCs] and 94 colorectal liver metastases [CRLMs]) in 172 patients treated with percutaneous MWA were retrospectively included. MAM of each tumor was assessed after expected ablation completion using intra-procedural CECT, allowing within-session additional ablation to any potentially insufficient margin. On immediate post-MWA MRI, complete ablation coverage of tumor and final MAM status were determined. The cumulative local tumor progression (LTP) rate was estimated by using the Kaplan-Meier method. To identify predictors of LTP, Cox regression analysis with a shared frailty model was performed.

**Results:**

Intra-procedural CECT findings prompted additional ablation in 18.9% (63/334) of tumors. Final complete ablation coverage of tumor and sufficient MAM were determined by MRI to be achieved in 99.4% (332/334) and 77.5% (259/334), and their estimated 6-month, 1-year, and 2-year LTP rates were 3.2%, 7.5%, and 12.9%; and 1.0%, 2.1%, and 6.9%, respectively. Insufficient MAM on post-MWA MRI, perivascular tumor location, and tumor size (cm) were independent risk factors for LTP (hazard ratio = 14.4, 6.0, and 1.1, *p* < 0.001, *p* = 0.003, and *p* = 0.011, respectively), while subcapsular location and histology (HCC vs CRLM) were not.

**Conclusions:**

In MWA of liver tumors, intra-procedural CECT monitoring of minimal ablative margin facilitates identification of potentially suboptimal margins and guides immediate additional intra-session ablation to maximize rates of margin-sufficient ablations, the latter being a highly predictive marker for excellent long-term local tumor control.

**Key Points:**

*• In MWA of liver tumors, intra-procedural CECT can identify potentially suboptimal minimal ablative margin, leading to immediate additional ablation in a single treatment session.*

*• Achieving a finally sufficient ablative margin through the MWA with intra-procedural CECT monitoring of minimal ablative margin results in excellent local tumor control.*

**Supplementary Information:**

The online version contains supplementary material available at 10.1007/s00330-022-08723-5.

## Introduction

Percutaneous thermal ablation therapy is one of major treatment options both for primary and metastatic liver tumors, such as hepatocellular carcinoma (HCC) and colorectal liver metastasis (CRLM). Radiofrequency ablation (RFA) is the most widely studied technique, while microwave ablation (MWA) is increasingly used as it allows larger ablation zone and shorter ablation time [1]. In ablation therapy, local tumor control is the primary goal which may improve long-term outcomes of patients. Accumulated evidence support that, for effective local tumor control, not only complete destruction of visible tumor on imaging, but creating a sufficient ablative margin in all dimensions is critical [2–5]. For now, widely accepted criteria for minimal ablative margin are ≥ 5 mm for HCC and ≥ 10 mm for CRLM, which have been reported to significantly lower local tumor progression (LTP) rates [6, 7].

Technical success of ablation treatment is routinely assessed on post-ablation contrast-enhanced CT (CECT) or MRI [8, 9], and repeat ablation session is performed based on the imaging results. However, considering the multi-step process from the initial MWA session to repeat treatment session, intra-procedural monitoring of treatment efficacy followed by additional ablation all within the same initial session has the potential to improve efficiency and patient convenience. In ablation therapy of liver tumors, B-mode ultrasound (US) is commonly used both for guiding and monitoring of the procedure. However, it has limitations as hyperechoic cloud of gas bubbles generated by ablation and the accompanying posterior acoustic shadow make accurate assessment difficult. In this regard, intra-procedural CECT can be a promising tool. With in-suite CECT with multi-planar reconstruction (MPR), 3D ablative margin status can be evaluated by comparing post-ablation images to pre-treatment images, which would not only help define tumors needing additional ablation, but also pinpoint exactly the margin where additional overlapping ablation may be required [10]. Until now, little is known about the added value of in-suite CECT imaging during liver tumor ablation in comparison to the conventional process which only includes post-ablation imaging.

This study aimed to investigate the usefulness of intra-procedural CECT-monitored minimal ablative margin control in MWA for successful ablation treatment of liver tumors.

## Materials and methods

This retrospective study was approved by the institutional review board, and met the requirements of the Declaration of Helsinki. The requirement of informed consent was waived.

### Study sample

From January 2017 to December 2019, patients who met the following criteria were enrolled in this study: (i) percutaneous liver MWA either for HCC or CLRM, (ii) MWA performed as a first-line treatment for the index tumor, without prior or combined loco-regional treatment such as trans-arterial chemoembolization, (iii) intra-procedural CECT after expected ablation completion of index tumor, (iv) available pre-treatment CT or MRI within 2 months from MWA, and (v) available immediate post-MWA MRI within 1 week. Tumors with difficulties determining post-MWA ablative margin status due to the following reasons were excluded: (i) aggregated or infiltrative index tumors on pre-treatment CT or MRI (difficult to delineate tumor boundary of each tumor), (ii) invisible tumor on pre-treatment CT or MRI, and (iii) suboptimal image quality of post-MWA MRI.

### Microwave ablation with intra-procedural CECT monitoring of minimal ablative margin

All MWA procedures were performed in an interventional suite with an in-suite CT scanner by one of three experienced radiologists (D.S.L., S.S.R, and J.P.M. with more than 5 years of experience in liver ablation therapy). Under general anesthesia, percutaneous MWA for liver tumor was done using 17- or 15-gauge gas-cooled tri-axial antennae (Certus Microwave System).

For each index tumor, the primary goal of MWA was to achieve a sufficient 3D ablative margin where technically feasible (i.e., where not restricted by distance to adjacent vessel ≥ 3 mm in diameter or liver capsule). Definition of sufficient ablative margin used in this study was ≥ 5 mm for HCC or ≥ 10 mm for CRLM at a minimum around tumor surface in accordance to prior studies [6, 7]. Intra-procedural CECT was performed after expected ablation completion to assess the margin status. If the ablative margin was assessed to be insufficient at any point circumferentially by intra-procedural CECT with MPR images, additional ablation was immediately performed targeted to that position. Regarding image registration to assess the ablative margin, in addition to visual (or “cognitive”) registration, software-based fusion technique was used in select cases based on availability and operator’s preference. Procedure details and intra-procedural CT protocol are provided in Supplements.

### Technical outcomes

Technical success of each index tumor was retrospectively evaluated by an abdominal radiologist (I.J. with 12 years of experience in liver imaging) blinded to whether an additional ablation had been performed during the MWA session. The term “session” is a synonym for procedure, which refers to a single MWA episode that consists of one or more ablations performed on one or more tumors [11]. Evaluations were performed on PACS comparing the immediate post-procedure MRI to the relevant pre-treatment CT and MRIs. For each index tumor, the radiologist determined whether (1) complete ablation coverage of the tumor and (2) sufficient minimal ablative margin were achieved. Post-MWA MRI protocol as well as details of how to assess the technical outcomes using MRI is indicated in Supplements.

### Local tumor progression outcomes

In tumors achieving complete ablation coverage based on immediate post-MWA MRI assessment, development of LTP was assessed during post-MWA follow-up. At our institution, post-MWA patients usually underwent follow-up liver CT or MRI at 1, 3, 6, 9, and 12 months and every 3–6 months thereafter. LTP was defined as the appearance of recurrent tumor foci in or along the ablation zone margin of MWA-treated lesions on CECT or MRI [12]. Tumors without LTP during the follow-up were censored at the last available assessment.

### Statistical analysis

Frequencies of within-session suboptimal minimal margin and additional ablation were calculated on a per-tumor basis. Baseline tumor characteristics, intra-procedural pre-post imaging comparison methods, and immediate technical success rates were compared between tumors with and without within-session additional ablation, using the Student *t* test for continuous variables and using the chi-square test for categorical variables. Tumor locations were categorized as either perivascular (a tumor abutting a portal vein or hepatic vein ≥ 3 mm in diameter) or non-perivascular; and either subcapsular (the distance between tumor margin and liver surface was < 10 mm) or non-subcapsular.

To evaluate the value of within-session additional ablation based on intra-procedural CT assessment of ablative margin, the estimated frequency of tumors with sufficient ablative margin when no additional within-session ablation was assumed for all tumors was compared to the actual frequency of tumors with sufficient ablative margin on immediate post-MWA MRI, using the McNemar test. The cumulative LTP rate on a per-tumor basis was estimated by using the Kaplan-Meier method. To identify predictors of LTP, univariate and multivariate Cox regression analysis with a shared frailty model was performed for the clustered data (variable number of tumors per patient).

All statistical analyses were performed on commercially available software (MedCalc® version 19.5.2: MedCalc Software and Stata version 15.0: StataCorp LLC). A *p* value of less than 0.05 was considered to indicate a statistical significance.

## Results

**Fig. 1:**
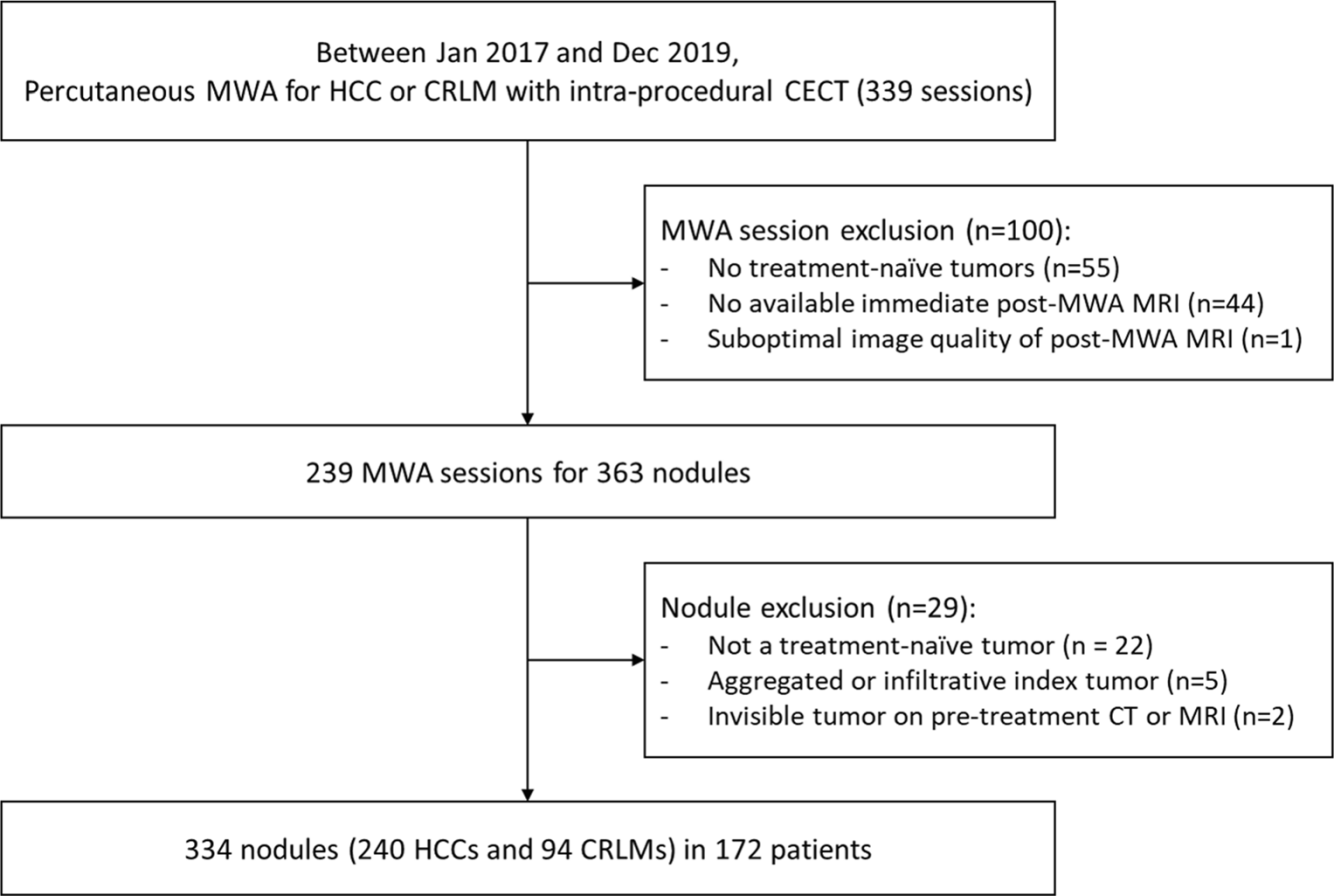
Patient flow diagram. MWA microwave ablation, HCC hepatocellular carcinoma, CRLM colorectal liver metastasis, CECT contrast-enhanced computed tomography

A total of 334 liver tumors in 172 patients were finally included in the analysis which were treated in 222 independent MWA sessions (Fig. 1). Index liver tumors included 240 HCCs and 94 CRLMs, of which 73 were perivascular and 235 were subcapsular, and although the majority of tumors were less than 30 mm in diameter, there were 41 tumors exceeding 30 mm. Consistent with the general indications for MWA, the tumors included in this study were all less than 50 mm with one exception, whereas one was larger than 50 mm on a retrospective measure (i.e., 57 mm). Patient and tumor characteristics are described in Table 1. There were 5 minor complications, including 3 small peri-hepatic hematomas, one transient pneumothorax, and one sub-segmental infarct.

**Table 1:** Characteristics of patients and liver tumors

Characteristics	Value (%)
Patients (*n* = 172)	
Age (years), mean ± standard deviation	65.5 ± 10.8 (range, 19–95)
Men:Women	128:44
Indications of MWA	
HCC	136 (79.1)
CRLM	36 (20.9)
Underlying chronic liver disease	
Chronic hepatitis B	26 (15.1)
Chronic hepatitis C	68 (39.5)
NAFLD/NASH	18 (10.5)
Alcoholic liver disease	15 (8.7)
Chronic liver disease of other causes	8 (4.7)
None	37 (21.5)
Number of MWA sessions per patient included in this study	
1	135 (78.5)
2	21 (12.2)
3 to 5	16 (9.3)
Tumors (*n* = 334)	
Size (mm), mean ± standard deviation	18.5 ± 8.9 (range, 2–57)
<20 mm	195 (58.4)
20–24 mm	67 (20.0)
25–29 mm	31 (9.3)
≥ 30 mm	41 (12.3)
Location	
Perivascular:Non-perivascular	73:261
Subcapsular:Non-subcapsular	235:99
Diagnosis	
HCC	240 (71.9)
CRLM	94 (28.1)

Data are number of patients or tumors with percentages in parentheses, unless otherwise specified. *HCC* hepatocellular carcinoma, *CRLM* colorectal liver metastasis, *MWA* microwave ablation, *NAFLD* non-alcoholic fatty liver disease, *NASH* non-alcoholic steatohepatitis

### Microwave ablation procedures

**Fig. 2:**
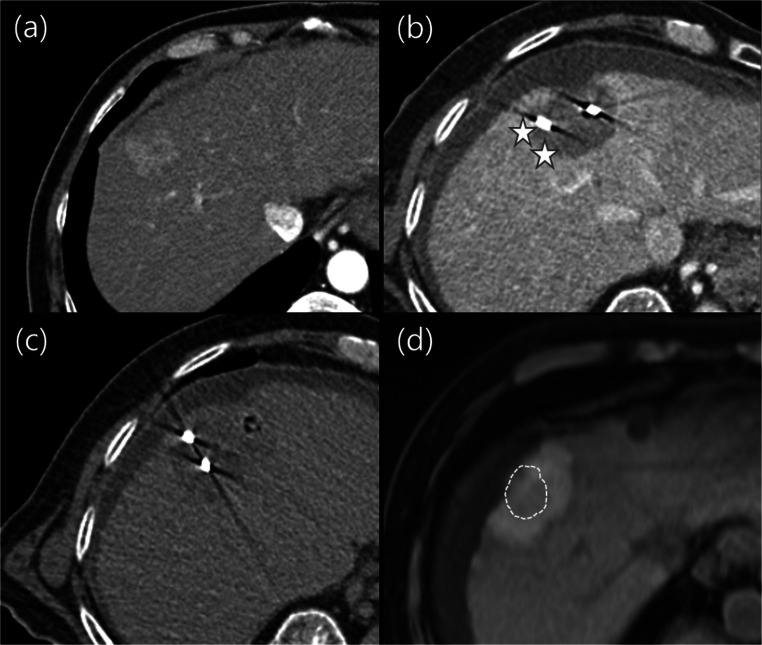
Patient with hepatocellular carcinoma (HCC) treated with microwave ablation (MWA). Pre-treatment arterial phase CT axial image (**a**) shows a hypervascular HCC at segment VIII of the liver. After expected ablation completion, ablative margin was assessed to be insufficient at the right side of tumor (stars) on intra-procedural CT images (**b**). Immediate additional ablation was performed by repositioning of MWA probes (**c**) to cover the site of insufficient margin. On post-MWA MRI, pre T1-weighted axial image (**d**) demonstrates that sufficient margin more than 5 mm is obtained around the tumor surface (dashed line) even at the right side of the tumor. Note that artificial ascites was introduced in this case due to subcapsular location of tumor and to minimize thermal injury to the diaphragm

Comparison of intra-procedural CECT after expected ablation completion to pre-treatment CT or MRI for minimal margin assessment was performed by visual registration alone in 68.6% (229/334) of tumors and by additional software-based fusion technique in 31.4% (105/334) of tumors. Based on the pre-post imaging comparison analysis, potentially suboptimal minimal margin was identified and immediate additional ablation was performed in 18.9% (63/334) of tumors (Fig. 2), while the other 81.1% (271/334) of tumors did not. Tumors requiring additional ablation (*n* = 63), in comparison to tumors without additional ablation (*n* = 271), were significantly larger in tumor diameter (mean 22.8 mm versus 17.6 mm, *p* < 0.001) and more frequently located in subcapsular locations (84.1% [53/63] versus 67.2% [182/271], *p* = 0.008). No significant differences were observed in frequencies of additional ablation according to the perivascular tumor location (perivascular versus non-perivascular, *p* = 0.077), intra-procedural pre-post imaging comparison methods (visual alone versus additional software-based registration, *p* = 0.207), or tumor diagnosis (HCC versus CRLM, *p* = 0.693) (Table 2).

**Table 2:** Comparison of tumors without versus with additional ablation after intra-procedural CT for minimal ablative margin assessment

Variables	Tumors without additional ablation (*n* = 271) (%)	Tumor with additional ablation (*n* = 63) (%)	*p* value^†^
Tumor size (mm), mean ± standard deviation	17.6 ± 8.2	22.8 ± 10.3	< 0.001*
< 20 mm	173 (63.8)	22 (34.9)	< 0.001*
20–29 mm	69 (25.5)	29 (46.0)	
≥ 30 mm	29 (10.7)	12 (19.0)	
Tumor location			
Perivascular	54 (19.9)	19 (30.2)	0.077
Subcapsular	182 (67.2)	53 (84.1)	0.008*
Tumor diagnosis			
HCC	196 (72.3)	44 (69.8)	0.693
CRLM	75 (27.7)	19 (30.2)	
Intra-procedural pre-post imaging comparison methods			
Visual (“cognitive”) registration only	190 (70.1)	39 (61.9)	0.207
Software-based registration added	81 (29.9)	24 (38.1)	
Technical outcomes based on immediate post-MWA MRI			
Complete ablation coverage of the tumor	269 (99.3)	63 (100)	0.495
Sufficient minimal ablative margin	205 (75.6)	54 (85.7)	0.085

Data are number of tumors with percentages in parentheses, unless otherwise specified. *HCC* hepatocellular carcinoma, *CRLM* colorectal liver metastasis, *MWA* microwave ablation, ^†^*p* values are calculated using the chi-square test for categorical variables and using the Student *t* test for continuous variables. *p values of statistical significance

### Technical outcomes

On immediate post-MWA MRI, complete ablation coverage of the tumor was confirmed in 99.4% (332/334) of tumors, while failed in two tumors (0.6%, 2/402) in two patients. Those two tumors that failed to ablate completely were invisible on in-suite pre-procedural CT while visible on pre-procedural MRI (< 10 mm in size) as well as post-MWA MRI.

Sufficient minimal ablative margin was achieved in 77.5% (259/334) of tumors according to the immediate post-MWA MRI, and the frequencies were trending toward but not significantly different between tumors treated without or with additional ablation (75.6% [205/271] versus 85.7% [54/63], *p* = 0.077) (Table 2). Since the tumors which received additional ablations by definition were suspected to have insufficient ablative margins at intra-procedure CECT, the estimated frequency of achieving sufficient ablative margin without margin-controlled additional ablations for all tumors was 61.4% (205/334). After additional ablation in 63 tumors, 54 tumors additionally achieved sufficient ablative margin on MRI, which resulted in the actual frequency of tumors with sufficient ablative margin of 77.5% (259/334). In other words, intra-procedural CECT with minimal margin assessment, by informing the need for additional ablation when necessary, played a decisive role in achieving sufficient ablative margin in an additional 16.2% (54/334) of tumors, which resulted in significant improvement in the per-tumor frequency of sufficient ablative margin (61.4% [205/334] versus 77.5% [259/334], *p* < 0.001).

### Local tumor progression outcomes

**Fig. 3:**
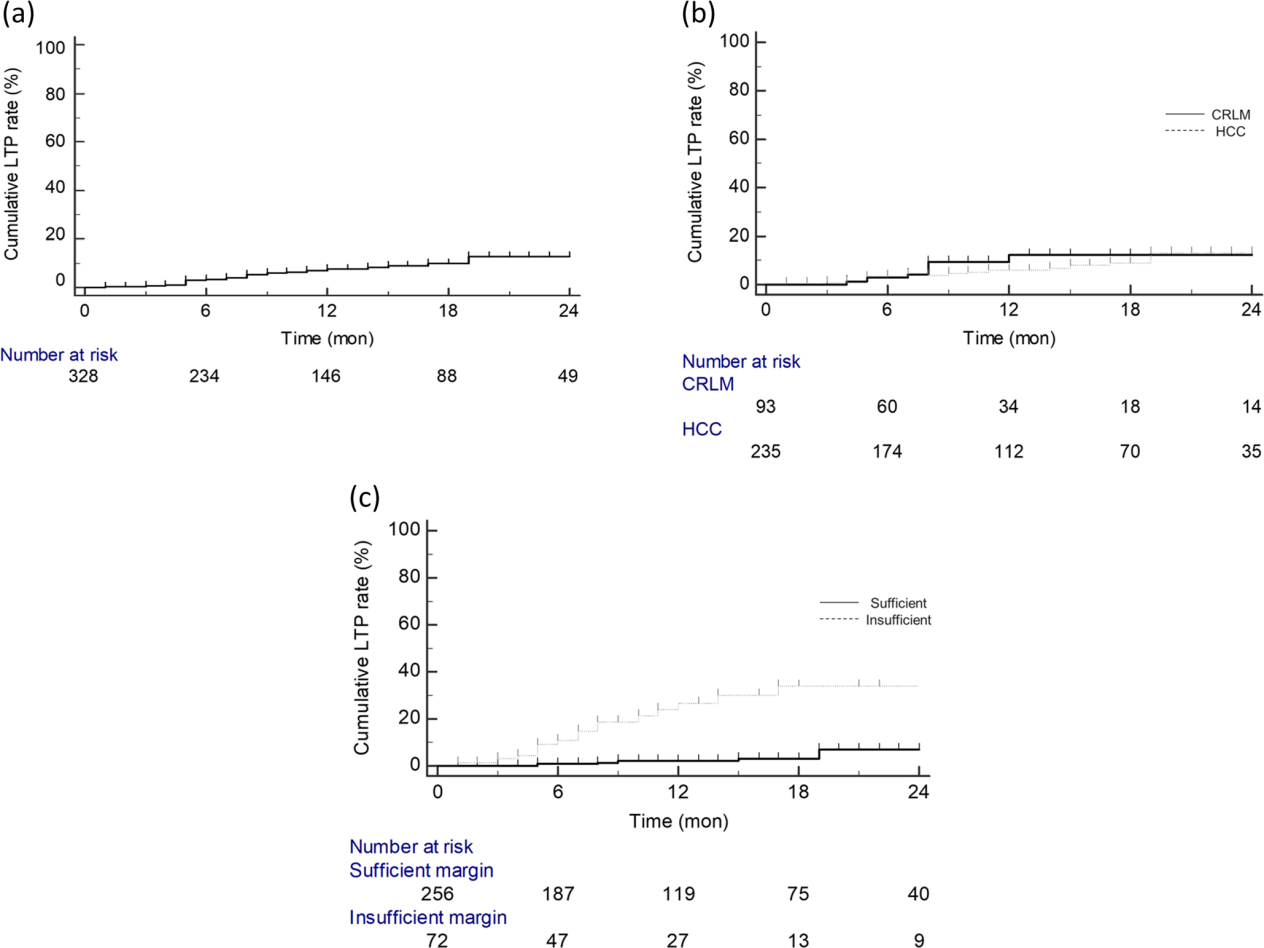
Cumulative incidence of local tumor progression after microwave ablation of all tumors (**a**), according to tumor diagnosis (**b**), and according to ablative margin status (**c**). LTP local tumor progression, HCC hepatocellular carcinoma, CRLM colorectal liver metastasis

Of tumors obtaining complete ablation coverage (*n* = 332), post-MWA follow-up data for LTP were available in 328 tumors (235 HCCs and 93 CRLMs) in 170 patients. Their median follow-up period was 11 months (range: 1–40 months). During the follow-up, LTP was detected in 25 tumors (7.6%, 25/328; 18 HCCs and 7 CRLMs). The estimated 6-month, 1-year, and 2-year LTP rates were 3.2%, 7.5%, and 12.9%, respectively, for all tumors; 3.4%, 6.0%, and 12.9% for HCCs; and 2.8%, 9.5%, and 12.0% for CRLMs (Fig. 3). According to minimal margin status on post-MWA MRI, estimated 6-month, 1-year, and 2-year LTP rates were 1.0%, 2.1%, and 6.9% for tumors with sufficient margin, and 10.9%, 26.6%, and 33.8% for tumors with insufficient margin (Fig. 3).

On univariate Cox regression analysis, insufficient ablative margin on post-MWA MRI and larger tumor size (cm) were significant risk factors for LTP (hazard ratio = 9.0 and 1.1; *p*s < 0.001) while tumor diagnosis (HCC versus CRLM), perivascular tumor location, and subcapsular tumor location were not (*p* = 0.709, 0.053, and 0.290, respectively). Multivariate Cox regression analysis revealed that insufficient ablative margin on post-MWA MRI, perivascular tumor location, and larger tumor size (cm) were independent risk factors for LTP (hazard ratio = 14.4, 6.0, and 1.1; *p* < 0.001, *p* = 0.003, and *p* = 0.011, respectively) (Table 3).

**Table 3:** Univariate and multivariate Cox regression analysis for factors associated with local tumor progression

Variables	Univariate analysis		Multivariate analysis	
	Hazard ratio (95% CI)	*p* value	Hazard ratio (95% CI)	*p* value
Tumor size (cm)	1.08 (1.05, 1.12)	< 0.001*	1.06 (1.01, 1.11)	0.011*
Tumor diagnosis of HCC (versus CRLM)	0.84 (0.34, 2.10)	0.709	1.60 (0.49, 5.23)	0.433
Perivascular location (versus non-perivascular)	2.24 (0.99, 5.09)	0.053	6.03 (1.83, 19.87)	0.003*
Subcapsular location (versus non-subcapsular)	1.71 (0.63, 4.62)	0.290	2.01 (0.57, 7.05)	0.274
Insufficient minimal ablative margin on post-MWA MRI (versus sufficient)	9.01 (3.72, 21.82)	< 0.001*	14.39 (4.61, 44.92)	< 0.001*

*CI* confidence interval, *HCC* hepatocellular carcinoma, *CRLM* colorectal liver metastasis, *MWA* microwave ablation, *p values of statistical significance

## Discussion

In local ablation treatment of liver tumors, intra-procedural imaging for margin monitoring could speed up the decision-making process of repeat ablation and thereby improve workflow. Our study demonstrated that intra-procedural CECT frequently directed within-session additional overlapping ablation (18.9% of tumors) by detecting suboptimal margins after MWA, ultimately yielding complete ablation coverage in 99.4% and sufficient minimal ablative margin (≥ 5 mm for HCC and ≥ 10 mm for CRLM) in 77.5% of tumors. This strategy provided good local tumor control outcomes, with an estimated 6-month, 1-year, and 2-year LTP rates of 3.2%, 7.5%, and 12.9%, respectively. Importantly, this outcome can be achieved in a single session, avoiding multi-session treatment courses.

Although other advanced imaging techniques such as real-time fusion imaging and contrast-enhanced US are being used for intra-procedural assessment of ablative margin [13], intra-procedural CT may have added advantages as it is known to be useful for guiding the ablation of tumors in challenging anatomical locations [14, 15]. In other words, the effectiveness of intra-procedural CT includes not only margin assessment, but also its ability to enable an accurate execution of additional ablation determined according to the assessment. Thus, in our study, the estimated frequency of tumors with sufficient margin at post-MWA MRI increased from 61.4 to 77.5%, quite high in comparison to prior studies for HCC [16] or CRLM [17], therefore supporting the concept of immediate CECT assessment during the procedure. According to our study results, tumors of larger size and subcapsular location showed higher frequency of insufficient ablative margin after an expected ablation completion, therefore requiring additional intra-session ablation. These results are in line with the general perception that larger tumor size and subcapsular location were factors that rendered percutaneous ablations technically more difficult. While MWA may have an advantage over RFA in treating larger tumors [18], full coverage of a large index tumor plus a sufficient 3D margin would still be challenging even with multiple probes. For subcapsular tumors, accurate tumor targeting can be difficult due to poor visibility by real-time US, and the probe location and energy application may need to be adjusted to prevent damage to adjacent structures [19]. Therefore, the use of intra-procedural CECT to confirm sufficient minimal ablative margin should be more actively considered for such technically challenging tumors.

In our study, local tumor control (i.e., 1-year estimated LTP rates of 7.5%) with single-session CECT-monitored MWA was comparable or superior to recent studies of MWA in liver malignancies [20–22]. In addition, our study confirmed ablative margin status as the most important independent predictor for LTP which highlights the importance of achieving sufficient minimal margin [13]. While the concept is not new, it is challenging to widely adopt due to the inherent difficulty in accurately assessing the ablative margin status by imaging during the ablation itself. At the very least a CECT would provide the best ablation zone boundary definition, but the index tumor boundary is no longer visible within the ablation zone. Therefore, the conventional method of visual registration of pre- and post-treatment images can be challenging and time-intensive. Recently, a software-based registration method has become available and was used based on operator discretion in our study population. As recent studies have shown the value of software-based registration for more sensitive detection of suboptimal margins [5, 16, 23], the full effect of software-based registration methods needs to be further tested in a prospective study.

It is notable that patients who ultimately did reach sufficient minimal margin, based on immediate MRI assessment, had very low rates of LTP (1.0%, 2.1%, and 6.9% at 6 months, 1 year, and 2 years). This low rate of LTP is rare in the published literature on percutaneous ablation of liver tumors, with the exception of a recent report using the technique of “no-touch” RFA, which ensures circumferential margin ablation with 3 electrodes enclosing a tumor but does not violate the tumor itself during the procedure [24]. In their series of “no-touch” RFA, LTP was virtually non-existent; however, their study population only included HCC averaging 1.6 cm and no more than 2.5 cm, therefore a super-selected group. For larger tumors and for tumors in subcapsular and perivascular locations, such enclosure techniques are often not technically applicable [24]. On the contrary, in our series, we had larger tumors (21.6% [72/334] exceeding 25 mm; including 41 tumors exceeding 30 mm), large number of subcapsular (235/334) and perivascular (73/344) tumors, and substantial number of CRLM (94/344). Therefore, we believe that in the end, for all tumors regardless of size, location, and histology, the ultimate equalizer is the pursuit of adequate minimal margins irrespective of ablation device, relying on operator skill to optimally position the applicators, and intra-procedural assessment to ensure full execution of the intended plan. In addition to ablative margin status, larger tumor size and perivascular tumor location were independent factors of LTP which were well correlated with prior studies of ablation treatment for liver malignancies [22, 25–28]. Of note, however, these features were not as strong an indicator of poor outcome as margin status, and the overall LTP rates in our study cohort despite including such tumors were very low, indicative of the power of a local tumor ablation strategy that primarily aims for true sufficient minimal ablation margins, while taking effective measures to compensate for larger tumor size and perivascular locations.

Our study had several limitations. First, as this study was of a retrospective design, the MWA protocol to determine additional ablation was not strictly controlled. Although there was general consensus between operators on a criterion of sufficient minimal ablative margin, i.e., ≥ 5 mm for HCC and ≥ 10 mm for CRLM, whether or not to perform additional ablation for each tumor was subjectively decided by considering margin status as well as other factors such as the risk of complication or expected efficacy of additional ablation. Prospective studies using detailed study protocols are needed to accurately validate the value of intra-procedural CECT. Second, in our study, post-MWA MRI was used as a reference standard for margin status, while intra-session additional ablation was determined based on CECT which may raise the concern about potential discrepancies for margin assessment depending on imaging modalities. However, our approach can be justified by the following reasons: CT can be applied quickly and conveniently for an intra-procedural use, and MRI is superior to CT in discriminating between index tumor and ablation margin [29]. Lastly, regarding treatment outcomes, only per-tumor results of LTP were analyzed in our study. Future studies with long-term follow-up data should reveal per-patient outcomes such as overall survival.

In conclusion, in MWA of liver tumors, achieving sufficient minimal ablation margins is highly predictive of local tumor control. Intra-procedural CECT monitoring of minimal ablative margin facilitates identification of potentially suboptimal minimal margins and guides immediate additional intra-session ablation, therefore optimizing local tumor control with a single-session treatment.

## Supplementary information

ESM 1 (DOCX 24 kb)

## Abbreviations

CECT: Contrast-enhanced computed tomography
CRLM: Colorectal liver metastasis
HCC: Hepatocellular carcinoma
LTP: Local tumor progression
MPR: Multi-planar reconstruction
MWA: Microwave ablation

## Declarations

### Guarantor

The scientific guarantor of this publication is David S. Lu.

### Statistics and biometry

One of the authors has significant statistical expertise.

### Methodology

• retrospective

• observational

• performed at one institution
