# Correction to: CT-monitored minimal ablative margin control in single-session microwave ablation of liver tumors: an effective strategy for local tumor control

**DOI:** 10.1007/s00330-022-08797-1

**Published:** 2022-05-10

**Authors:** Ijin Joo, Kenneth W. Morrow, Steven S. Raman, Justin P. McWilliams, James W. Sayre, David S. Lu

**Affiliations:** 1 Department of Radiology, David Geffen School of Medicine, University of California, Los Angeles, CA USA; 2 Department of Radiology, Seoul National University Hospital and Seoul National University College of Medicine, Seoul, South Korea

**Correction to: European Radiology**

10.1007/s00330-022-08723-5

The original version of this article, published on 7 April 2022, unfortunately contained a mistake. In the column '*p* value†' of Table 2, some data was mistakenly repeated. The original article has been corrected.

**Table 2:** Comparison of tumors without versus with additional ablation after intra-procedural CT for minimal ablative margin assessment

Variables	Tumors without additional ablation (*n* = 271) (%)	Tumor with additional ablation (*n* = 63) (%)	*p* value†
Tumor size (mm), mean ± standard deviation	17.6 ± 8.2	22.8 ± 10.3	< 0.001*
< 20 mm	173 (63.8)	22 (34.9)	< 0.001*
20–29 mm	69 (25.5)	29 (46.0)	
≥ 30 mm	29 (10.7)	12 (19.0)	
Tumor location			
Perivascular	54 (19.9)	19 (30.2)	0.077
Subcapsular	182 (67.2)	53 (84.1)	0.008*
Tumor diagnosis			
HCC	196 (72.3)	44 (69.8)	0.693
CRLM	75 (27.7)	19 (30.2)	
Intra-procedural pre-post imaging comparison methods			
Visual (“cognitive”) registration only	190 (70.1)	39 (61.9)	0.207
Software-based registration added	81 (29.9)	24 (38.1)	
Technical outcomes based on immediate post-MWA MRI			
Complete ablation coverage of the tumor	269 (99.3)	63 (100)	0.495
Sufficient minimal ablative margin	205 (75.6)	54 (85.7)	0.085

Data are number of tumors with percentages in parentheses, unless otherwise specified. *HCC* hepatocellular carcinoma, *CRLM* colorectal liver metastasis, *MWA* microwave ablation, † *p* values are calculated using the chi-square test for categorical variables and using the Student *t* test for continuous variables. *p values of statistical significance

