# Supplementary material for: CT-monitored minimal ablative margin control in single-session microwave ablation of liver tumors: an effective strategy for local tumor control
Source: Eur Radiol. 2022 Apr 7;32(9):6327–35. doi: 10.1007/s00330-022-08723-5 (PMC9381632; doi:10.1007/s00330-022-08723-5)
Supplement: Supplementary file 1 — (DOCX 24 kb) [file 330_2022_8723_MOESM1_ESM.docx]

**Procedure details of MWA with intra-procedural CECT monitoring of minimal ablative margin**

To guide probe placement, both US and CT were used. In the majority of cases, each microwave probe was inserted under real-time US guidance and its position was also verified by CT. Hydro-displacement technique prior to ablation was optionally used to avoid thermal injury for tumors abutting diaphragm, stomach, or bowel. According to tumor size and location, ablations using single or multiple overlapping probes were performed at the operator’s discretion. After expected ablation completion, immediate CECT was performed to monitor the treatment. This may be done with the microwave antenna left in situ or after complete withdrawal, the former being preferred due to ease of repositioning of the antenna(s) for additional ablations as necessary. By comparing post-ablation CECT images to pre-treatment imaging studies (prior CT or MRI as well as in-suite pre-procedure CT for tumor localization), operators determined whether a sufficient minimal ablative margin was achieved, and if not, pinpointed the location of the insufficient minimal margin. In all cases, imaging evaluation was done using visual (or “cognitive”) registration using side-by-side comparisons on a PACS (Picture Archiving and Communication System) workstation. Additional software-based fusion technique (NeuWave Ablation Confirmation Software) was used in select cases based on availability and operator’s preference. If sufficient minimal ablative margin was not achieved after careful assessment of the pre-treatment and intra-procedure CECT images, probes were repositioned to the position of potentially suboptimal margin and additional ablation was performed, and this process was documented in the procedure report.

**Intra-procedural CT protocol**

All intra-procedural CT exams were performed with a 64-channel scanner (SOMATOM Definition: Siemens Healthineers). For CT acquisition parameters, in general a beam collimation of 0.625 mm x 64, a pitch of 0.75, a gantry rotation time of 0.75 sec, and kVp/effective mA of 120/200 was used.

Intra-procedural CT exams for initial tumor localization were done either with or without use of intravenous contrast, determined by the operator depending on tumor visibility and conspicuity at non-contrast CT and at B-mode US. After expected ablation completion, CECT was performed: portal venous phase in CRLM patients; late arterial phase and portal venous phases in HCC patients, and CECT images were reconstructed in axial, coronal, and sagittal planes at 2-3 mm for minimal margin assessment. Intermittent CT images for confirmation of probe position during lesion targeting did not require any contrast media.

**Immediate post-ablation MRI protocol**

At our institution, after MWA of liver tumors, gadoxetic acid-enhanced liver MRI within 24 hours is routinely performed. However, due to variability of patient needs and MRI availability, MRI taken within 1 week after the treatment was considered an immediate follow-up exam in this study. Liver MRIs were obtained on either 1.5T or 3T scanners. MRI sequences included axial images of heavily T2-weighted imaging, T2-weighted fast spin echo imaging, dual echo gradient echo imaging, diffusion-weighted imaging, and pre and post-contrast fat suppressed T1-weighted images. Post-contrast T1-weighted images included arterial phase, portal venous phase, transitional phase, and hepatobiliary phase after intravenous administration of 0.1 mmol/kg of gadoxetic acid. Coronal images of portal venous phase and hepatobiliary phase were also obtained in addition to the axial images of each phase, respectively.

**Assessment of technical outcomes on post-ablation MRI**

Complete ablation coverage of the tumor indicated no visible residual tumor within or at the periphery of the ablation zone on MRI, regardless of ablative margin beyond tumor surface. Sufficient ablative margin was defined as ≥5 mm for HCC or ≥10 mm for CRLM around tumor surface, equivalent to the definition used in intra-procedure assessment. However, if the distance from tumor to liver capsule or a patent vessel is less than the 5 mm or 10 mm, achieving ≥5 mm or ≥10 mm ablative margin at these positions is technically impossible. Thus, the distance criteria were not applied to margins points next to adjacent vessel (≥3 mm in diameter) or liver capsule. Actual margin assessment was facilitated by simultaneous identification of the ablation zone boundary and the underlying tumor boundary, latter often visible on T1W images [1, 2]. In cases where underlying tumor boundary was not visible within the ablation zone on MRI, the ablative margin was therefore derived by detailed visual registration and comparison between pre-treatment tumor boundary and post-treatment ablation zone boundary relative to surrounding landmarks. If the tumor boundary was not visible, potential tumor shrinkage associated with the ablation was not considered in margin assessment because the shrinkage degree in all dimensions could not be accurately and objectively predicted.

**REFERENCES**

1. Takeyama N, Vidhyarkorn S, Chung DJ et al (2016) Does hepatobiliary phase sequence qualitatively outperform unenhanced T1-weighted imaging in assessment of the ablation margin 24 hours after thermal ablation of hepatocellular carcinomas? Abdom Radiol (NY) 41:1942-1955

2. Koda M, Tokunaga S, Miyoshi K et al (2012) Assessment of ablative margin by unenhanced magnetic resonance imaging after radiofrequency ablation for hepatocellular carcinoma. Eur J Radiol 81:2730-2736
